# Supplementary material for: Venomix: a simple bioinformatic pipeline for identifying and characterizing toxin gene candidates from transcriptomic data
Source: PeerJ. 2018 Jul 31;6:e5361. doi: 10.7717/peerj.5361 (PMC6074769; doi:10.7717/peerj.5361)
Supplement: Supplemental Information 3 [file peerj-06-5361-s003.gz › FinalOutput_E-6/Thrombin-like_enzyme_catroxobin-1_1/finaltree.pdf]

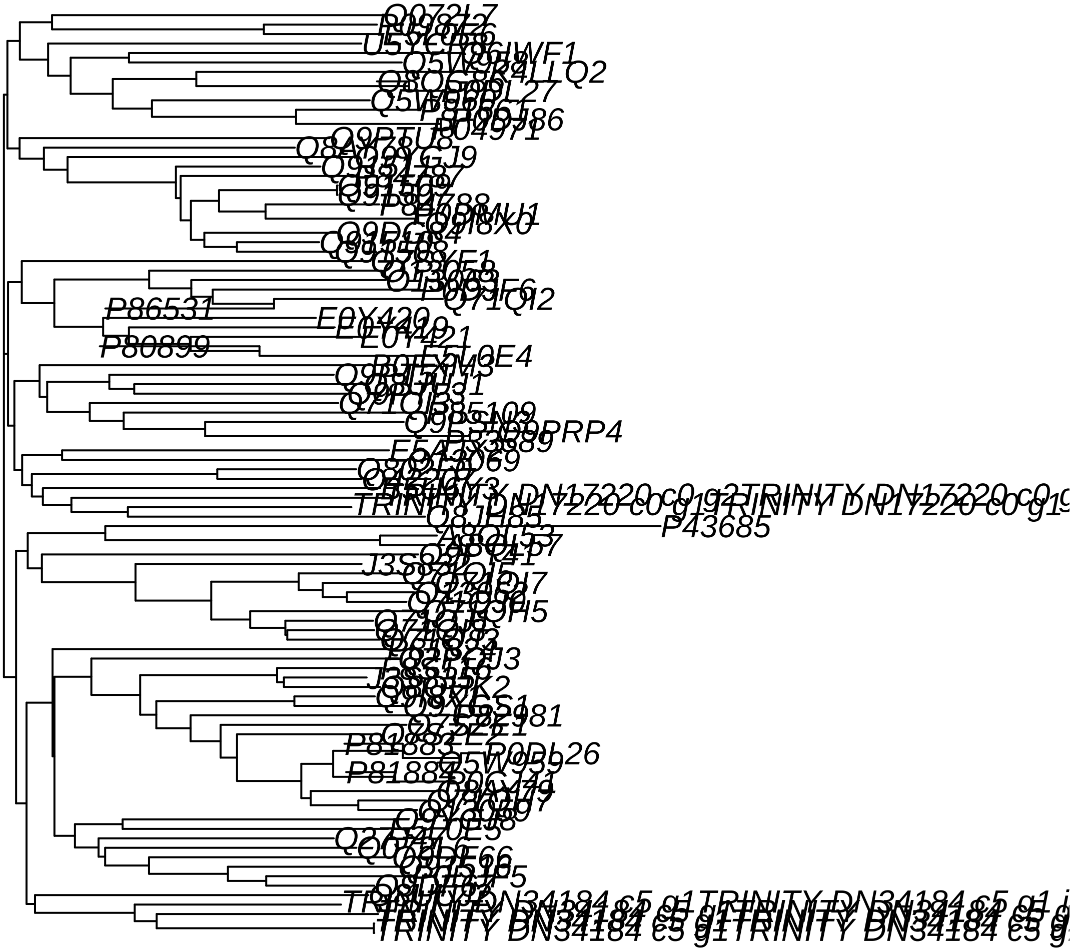
P86531
P80899
P43685
P81883
P81884
Q71QI2
E0Y420
Q0PRP4
